# Industrial equipment detection algorithm under complex working conditions based on ROMS R-CNN

**DOI:** 10.1371/journal.pone.0266444

**Published:** 2022-04-07

**Authors:** Junpeng Wu, Shaobo Tang, Xianglei Li, Yibo Zhou

**Affiliations:** 1 Key Laboratory of Modern Power System Simulation Control and Green Power New Technology of Ministry of Education, Northeast Electric Power University, Jilin, China; 2 School of Electrical Engineering, Northeast Electric Power University, Jilin, China; 3 The People’s Bank of China JiLin Central Sub-Branch, Jilin, China; University Tunku Abdul Rahman, MALAYSIA

## Abstract

In the paper, we proposed a deep learning-based industrial equipment detection algorithm ROMS R-CNN (Rotation Occlusion Multi-Scale Region-CNN). It can solve the problem of inaccurate detection of industrial equipment under complex working conditions such as multi-scale ratio, rotation tilt, occlusion and overlap. The method proposed in this paper first is to construct the MobileNetV2 as the feature pyramid network, and then to combine high semantic information with high resolution information solved industrial equipment detection of different scales. Secondly, a specific rotation anchor scheme is proposed, and the data set is clustered through the k-means algorithm to obtain a specific aspect ratio. Combined with the rotation angle, a rotation anchor of any direction and size is generated to solve the problem of easy tilting of industrial equipment. Finally, a Non-Maximum Suppression algorithm with penalty factors is introduced to solve the overlapping in industrial equipment detection. The experimental results in common industrial equipment detection show that this method is better than other algorithms, significantly improves the missed detection and false detection, and the mAP reaches 0.939.

## 1 Introduction

At present, noncontact detection technology based on computer vision plays a crucial role in the field of industrial equipment inspection. In the process of manual inspection in the industrial equipment area, except for time-consumption, wide unpredictable range of problems will be involved mainly due to the location and angle of the industrial equipment. Specifically, the actual shortcomings include: the first most dangerous factor for the safety operation of manual inspection is generally from the location of industrial equipment, harsh environment, and the existence of medium of the harmful, flammable and explosive gas and liquid; the second is low efficiency caused by repeatedly viewing the same places, which results in the visual and mental fatigue; the third is attributed to the high error rate from manual inspections of industrial equipment. The practical implementations of convolutional neural networks with proven effectiveness on large-scale object recognition tasks can improve industrial equipment inspection techniques, especially when leveraging other “big data” best practices and tools. Such inspection methods can provide historical records (image databases) for trending and analysis of instrument readings and preventative / predictive maintenance; replace manual on-site inspections with their potential human error, freeing up personnel for more difficult tasks; help realize the potential for automation in industries such as chemical factories. The use of deep learning methods can reduce or eliminate the need for specifically designed makers and fiducials for equipment location and detection as well, allowing easy retrofits of existing legacy equipment and saving costs. The rotating occlusion multi-scale region CNN (ROMS-R-CNN) detection method proposed in this paper can optimize and improve the identifying speed of location and accuracy of detection on different scales, aspect ratio, rotation tilt and occlusion of industrial equipment. Our contributions are as follows:

1. The proposed MobilNetV2 FPN network based on the MobilNetV2 network, which is suitable for actual industrial sites for its lightweight network, can detect industrial equipment at multiple scales.
2. A specific rotary anchor scheme is proposed, which can be used to detect industrial equipment with different aspect ratios and different rotation tilt angles.
3. A multi-stage penalty non-maximum suppression algorithm is proposed, which has a good detection effect for industrial equipment with multi-occlusion overlapping.
4. Three categories of industrial equipment images are collected and expanded through data enhancement. The final experiment shows that the proposed algorithm has an improved effect on the detection of industrial equipment and provides a reference for the research of object detection, automatic inspection and industrial automation of industrial equipment in the future.

## 2 Related work

In 2013, the object detection of Object Proposal based on the R-CNN series of deep learning was proposed. Subsequently, the optimized SPP Net [1] and Fast R-CNN [2] were successively proposed in 2014 and 2015. Faster R-CNN [3] solved the problem of still needing to use external algorithms to extract candidate boxes, thus realizing end-to-end processing. In addition, YOLO [4–6] series and SSD series [7–9] based on convolutional integrated networks have also been proposed and improved continuously. Faster R-CNN is very effective for ordinary object detection, but it is somewhat difficult to detect small objects. As in [10] a feature pyramid network (FPN) was proposed to improve the detection of multi-scale objects by combining low-level location information and high-level semantic information. The author of [11] proposed the small object detection in optical remote sensing images based on modified Faster R-CNN. The method not only modifies the RPN stage of Faster R-CNN by setting appropriate anchors but also leverages a single high-level feature map of a fine resolution by designing a similar architecture with top-down and skip connections. The authors of [12] used the feature pyramid network in the SSD detection algorithm based on the convolutional integrated network, and the effect was also verified in the VOC data set. Literature [13–17] also adopted this method to make different efforts for small object detection. Subsequent Mask R-CNN [18] added another branch on the basis of Faster R-CNN to increase an output, that is, the object mask, which changed from the original two tasks (classification and regression) to three tasks (classification, regression and segmentation). In addition, in terms of rotation, R2CNN [19]and RRPN [20] are both for the detection of rotated text scenes, but the negative impact of non-maximum suppression still exists due to the used horizontal anchors of R2CNN in the first stage. Subsequently, RRPN adopted rotating anchor points, which effectively improved the quality of regional proposals. In literature [21], a new method of RBox detection is proposed. The DRBox is being tested to detect ships and aircraft on satellite images. DRBox is designed as a box-based approach, possible to apply RBox to a recommended detection framework [22–25] on rotation detection, and many of them are for ship detection. The authors of [26] proposed an automatic ship detection method RDFPN for the rotating dense feature pyramid network of remote sensing objects, which has a significant effect on automatic ship detection. In literature [27], a new detection model based on multi-task rotation region was proposed to solve the complexity of application scenes in remote sensing field. The author of [28] proposed SCRDet, a multi-category rotary detector. The monitored pixel attention network and channel attention network were explored for small and cluttered object detection. The author of [29] proposed an insulator orientation recognition algorithm based on deep learning, which is based on SSD and can detect insulators when they are tilted. The authors of [30–31] proposed an improved algorithm for non-maximum suppression post-processing algorithm, which has a certain improvement to the missed detection under certain conditions. On the other hand, the authors of [32] proposed a lightweight and deep convolutional separable network structure model, which reduces a large number of calculation parameters while ensuring that the processing accuracy does not drop significantly. The authors of [33] made a further improvement on this basis to optimize the model structure, by referring to the advantages of the residual network, and proposed the inverted residuals block structure, which is also a lightweight network. The authors of [34] first proposed the use of convolutional neural network YOLO to identify and locate industrial equipment valves, and achieved good results, but it was only single-classification valve detection. The authors of [35] didn’t mention the detection of the level gauge that uses intelligent algorithms to perform intelligent readings on the meter. The method proposed in this paper can detect multiple types of industrial equipment and with improved results.

## 3 Proposed method

### 3.1 Network structure

**Fig 1:**
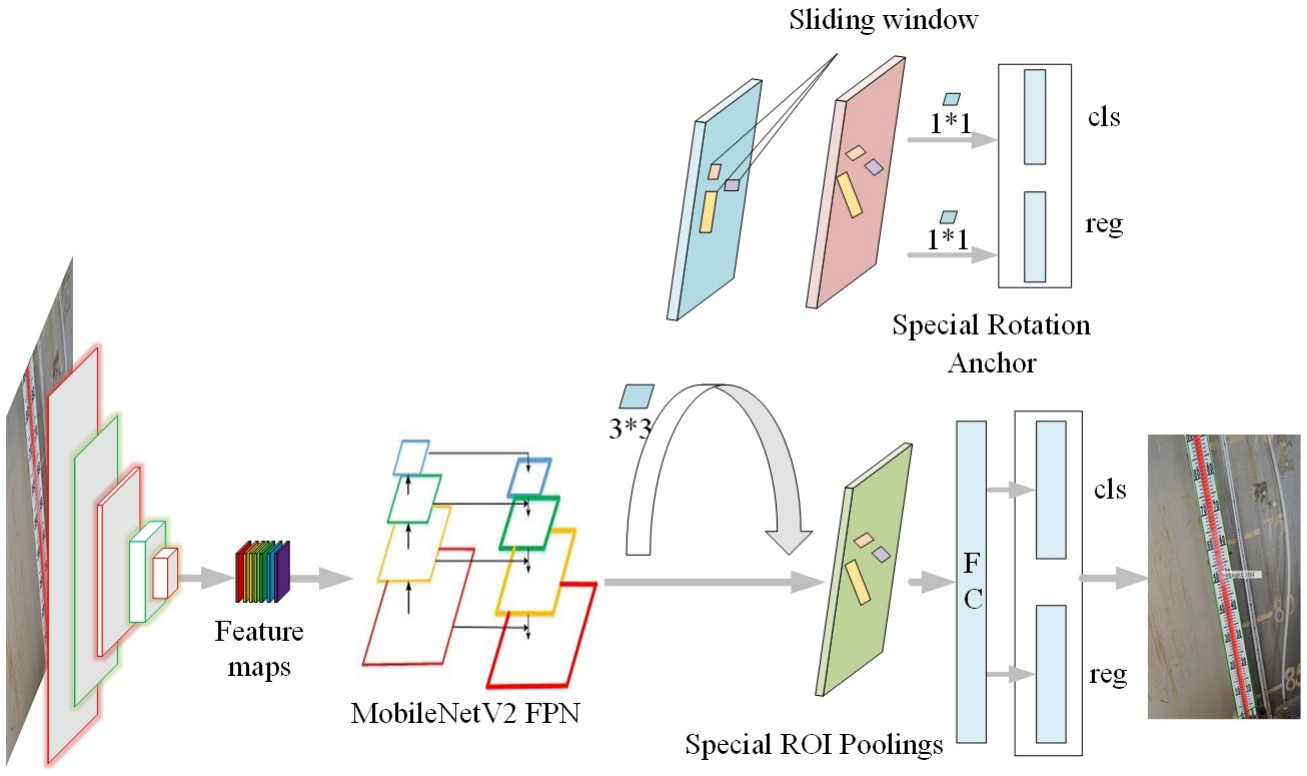
ROMS R-CNN network structure. An inspection example of level gauge is shown in Fig 1. The left structure is a convolutional layer set that produces a set of feature maps by the MobileNetV2 FPN. Sliding through the sliding window can produce anchors of different sizes and rotation angles. Special ROI pooling layers are used to unify the size of candidate areas. As a result, we can get the identified and located level gauge.

The structure flow chart of ROMS R-CNN proposed in this paper is shown in Fig 1. First of all, the input image passes through the MobileNetV2 FPN feature extraction network to obtain the initial feature map. After up-sampling, the initial feature map are connected and fused horizontally to obtain multi-scale feature map, and then to pass Region Proposal Network (RPN) for the generation of the anchor. Region proposal can be obtained after anchor is dichotomized in classification layer (cls) and preliminarily located in regression layer (reg). Then, region proposals are normalized by the pooling layer of the Region of Interest (ROI). Finally, the proposal box with the highest confidence is outputted through the Full Connection layer (FC), multi-classification layer, accurate boundary box regression of regression layer and non-maximum suppression algorithm of multi-stage punishment.

### 3.2 MobileNetV2 FPN network

At present, the ResNet series of networks with better detection results have many layers to cause excessive calculations, and excessive complexity leads to more delays. At the same time, hardware devices are required to have strong calculations and storage capabilities. However, in actual industrial equipment testing, many factories do not have such hardware supporting conditions, which makes it difficult to apply such large and complex models. In addition, during the actual industrial equipment detecting, various industrial equipment can be encountered, including the equipment within the size from meter to centimetre scale. At the same time, the distance of taking pictures will also affect the scale of industrial equipment image.

**Fig 2:**
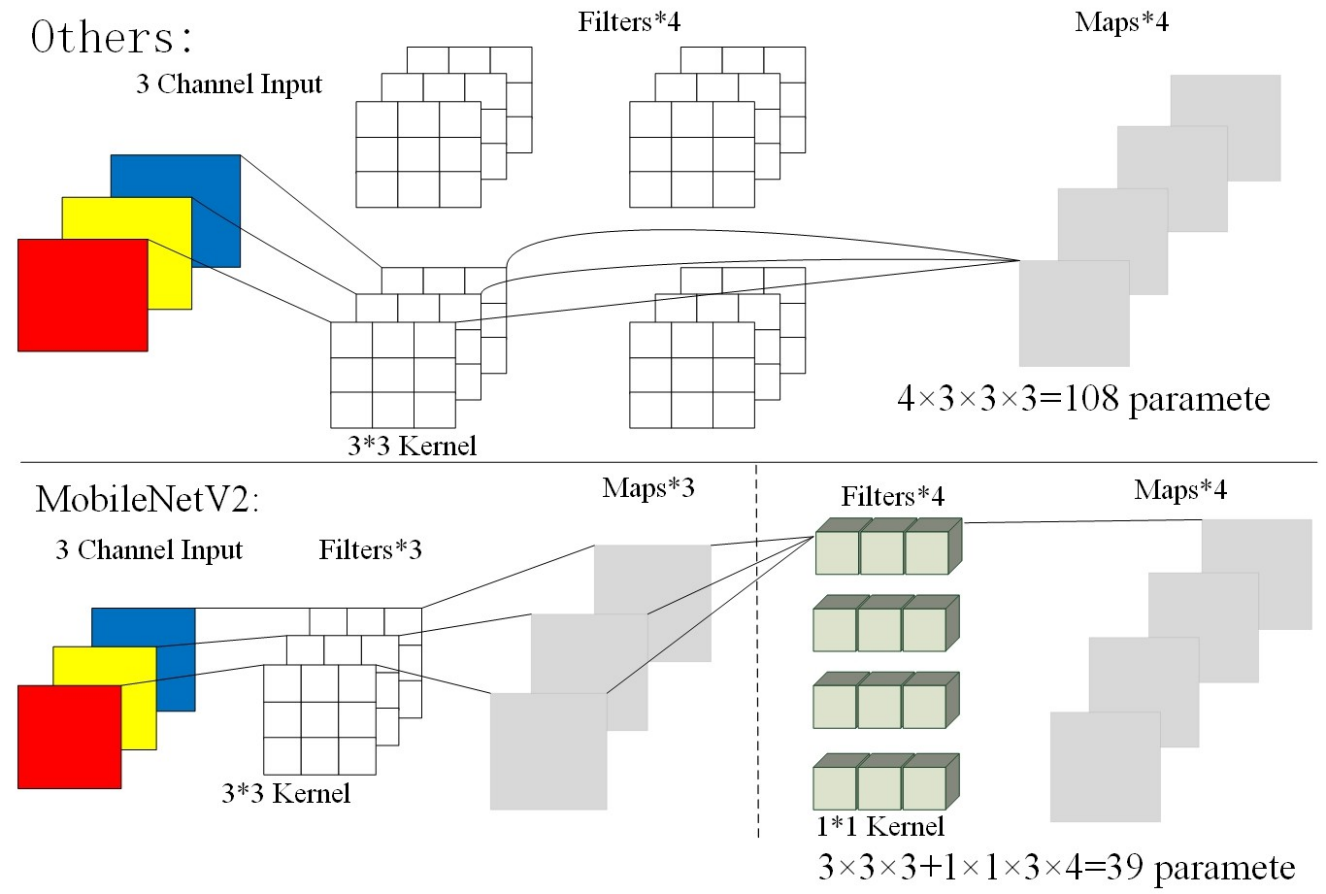
The parametric comparison between the MobileNetV2 network and the rest of the network.

In response to the above problems, we reconstructed a lightweight network to detect multi-scale industrial equipments. The MobileNetV2 network is transformed into a feature pyramid structure, which combines the advantages of a deep convolutional separable structure and a feature pyramid structure solved the problems of multi-scale detection and time delay in the actual detection. The basic unit of MobileNetV2 is a separable depthwise convolution. The calculation of the convention is divided into two parts. the first is to perform depthwise convolution on the channels, and stitch the output. The second is to use the unit convolution kernel to perform pointwise convolution for the feature map. The overall effect is similar to the standard convolution, but it will greatly reduce the calculation parameters, making the calculation amount to 1/n of the original, and n is the size of the convolution kernel. A comparison of MobileNetV2 parameters with other networks is shown in Fig 2. Choosing the MobileNetV2 network can ensure accuracy while reducing memory usage, and also reduce time overhead. As shown in Table 1, the size and complexity of operation parameters of several different network structures are compared in literature [33], and it can be seen that the amount of calculation of MobileNetV2 is small. In this paper, we also compare the detection time of two networks MobileNetV2 and ResNet-50. MobileNetV2 took 0.435s and ResNet-50 0.614s. The short time of MobileNetV2 can also prove that it has a small amount of calculation and low complexity.

**Table 1:** Comparison of parameters and complexity of different network structures in feature extraction.

Network	Params	MAdds
YOLOV2	50.7M	12.5B
ResNet-101	58.16M	81.0B
MobileNetV1	11.15M	14.25B
MobileNetV2	4.52M	5.8B

**Fig 3:**
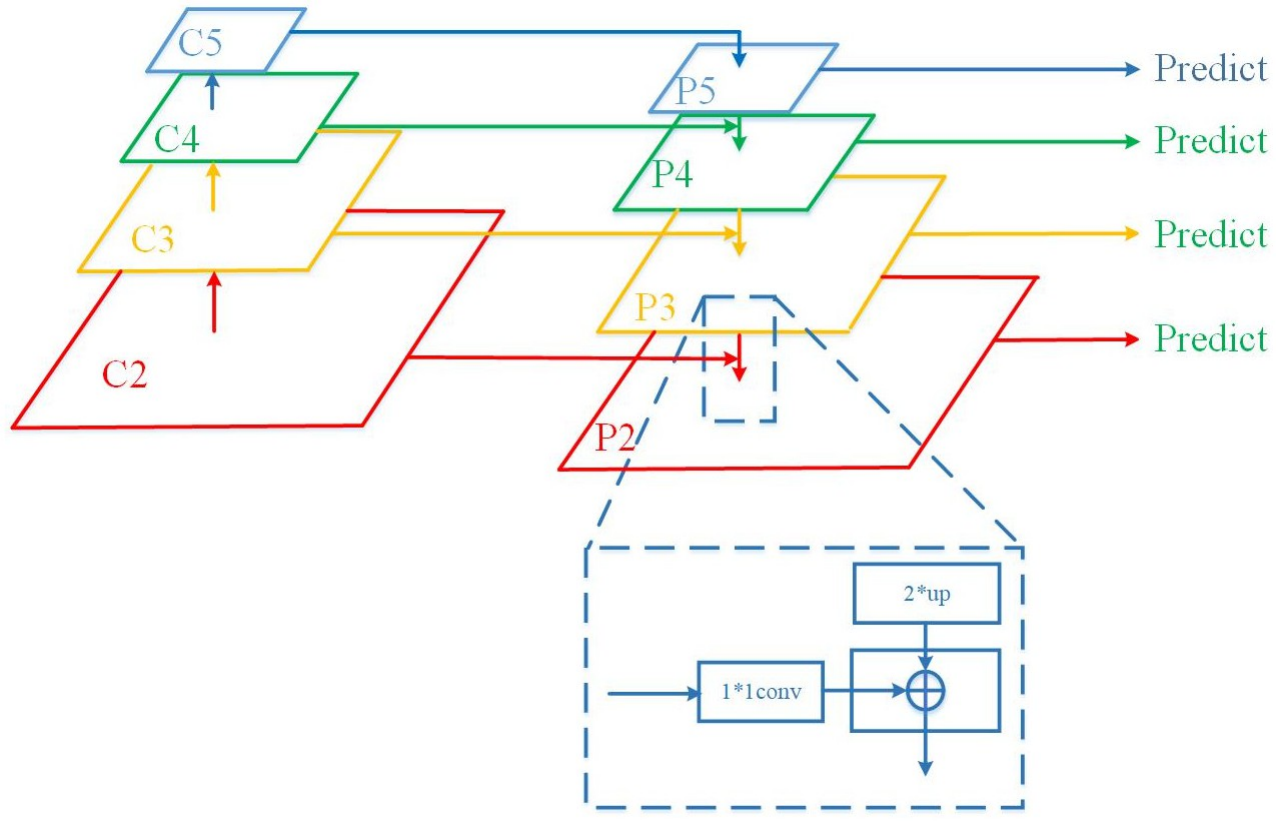
MobileNetV2 FPN structure.

The result of network construction is shown in Fig 3. It consists of three structures. the first is a bottom-up down-sampling structure, the last layer of 4 inverted residual blocks is selected in MobileNetV2, named C2, C3, C4, C5, to generate the pyramid structure for the higher resolution. The second is the top-down up-sampling structure, making C5 convolved by 1*1 as the top layer P5 of the feature pyramid, carrying out double up-sampling, and then fusing with C4 to get P4. In this way, P3 and P2 are obtained successively, which are mainly used to provide due to the upper level of the pyramid. There is also a horizontal connection that combines the result of up-sampling with the feature map of the same size generated from the bottom up. First, C5, C4, C3, and C2 are subjected to 1*1 convolution to reduce the number of channels. Then, it goes through a 3*3 convolution to mitigate aliasing effects. In this way, a MobileNetV2 FPN network can combine the feature mapping of low resolution and strong semantic information with the feature of high resolution and weak semantic information. Each layer contains strong semantic information, and each layer can be predicted separately.

### 3.3 Specific rotation anchor

RPN is the core component of ROMS R-CNN. The feature mapping output by the MobileNetV2 FPN network is slidden by a 3*3 sliding window to generate an anchor. Since these anchors generally use a fixed aspect ratio, it is not suitable for industrial equipment with different aspect ratios or extremely large aspect ratios.

Therefore, we proposed a specific rotation anchor scheme. In this scheme, K-means clustering algorithm is to pre-select the industrial equipment data set to find the optimal aspect ratio and then combine with the rotation angle for the generation of anchors. It will provide the industrial equipment detection with both higher length-to-width ratio and wider rotation angle. The bounding box is properly modified through the network. Finally, we get precise positioning.

For the k-means clustering algorithm, if the Euclidean distance criterion is used, the larger bounding box will produce more errors than the smaller one. Therefore, IoU is adopted for calculation, and the distance evaluation criteria is shown in Eq (1).


$$D({\text{B}}_{\text{1}},{\text{B}}_{\text{2}})=1-\text{IoU}({\text{B}}_{\text{1}},{\text{B}}_{\text{2}})$$ (1)

**Fig 4:**
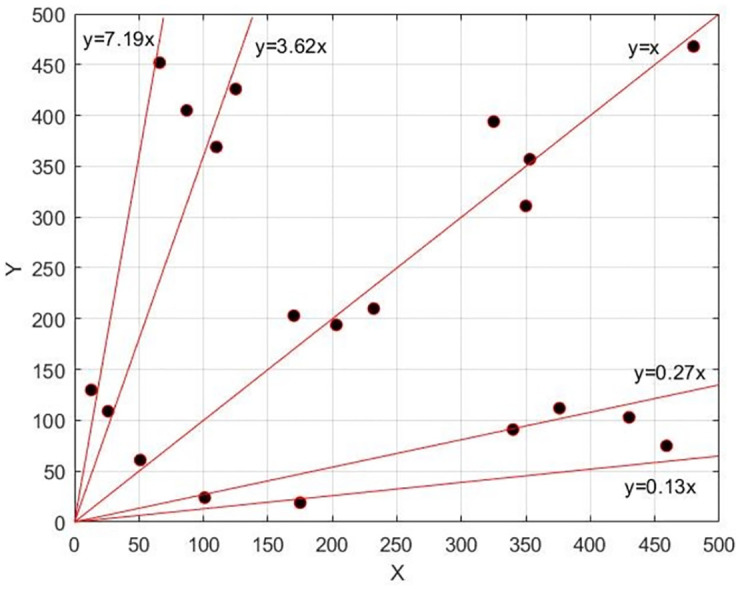
Scatter diagram of aspect ratio of anchor.

Here, B_1_ is the default anchor frame; B_2_ is the object reality box. The higher IoU makes the smaller error D, and the increasing number of anchor frames can provide the clustering with higher accuracy, but this will result the increase of this clustering complexity. In order to consider the accuracy of clustering and the complexity of the algorithm, we set the number of anchor frames as 20, calculate the error between each anchor frame and each real frame, assign the real frame to the anchor frame with the smallest error successively, recalculate the length and width of the anchor frame according to the real frame, repeat the above operation until the length and width of the anchor frame no longer change, and finally get the output as shown in Table 2. According to Table 2, a scatter plot is drawn in the form of coordinate points as shown in Fig 4. Through linear regression, five linear functions with different slopes are obtained, and the slopes (0.13, 0.27, 1, 3.62, 7.19) are the special aspect ratio.

**Table 2:** The results of aspect ratio obtained after k-means clustering.

H	W
51	61
203	194
353	357
480	468
232	210
170	203
325	394
350	311
430	103
459	75
66	452
101	24
125	426
340	91
175	19
13	130
87	405
110	369
26	109
376	112

In the Table 2, H represents the height of the object and W represents the width of the target. This is not the actual width and height, but the proportion one. The Table 2 shows the 20 groups of different width-to-height ratio results obtained by clustering. According to these results, five width-to-height ratios can be obtained by regression.

The traditional detection box is the horizontal or vertical rectangle, determined by the coordinates of the two points at the upper left and the lower right corners, namely four parameters (x_1_, y_1_, x_2_, y_2_). However, it does not contain angle information and cannot solve the problem of rotation and tilt of industrial equipment.

**Fig 5:**
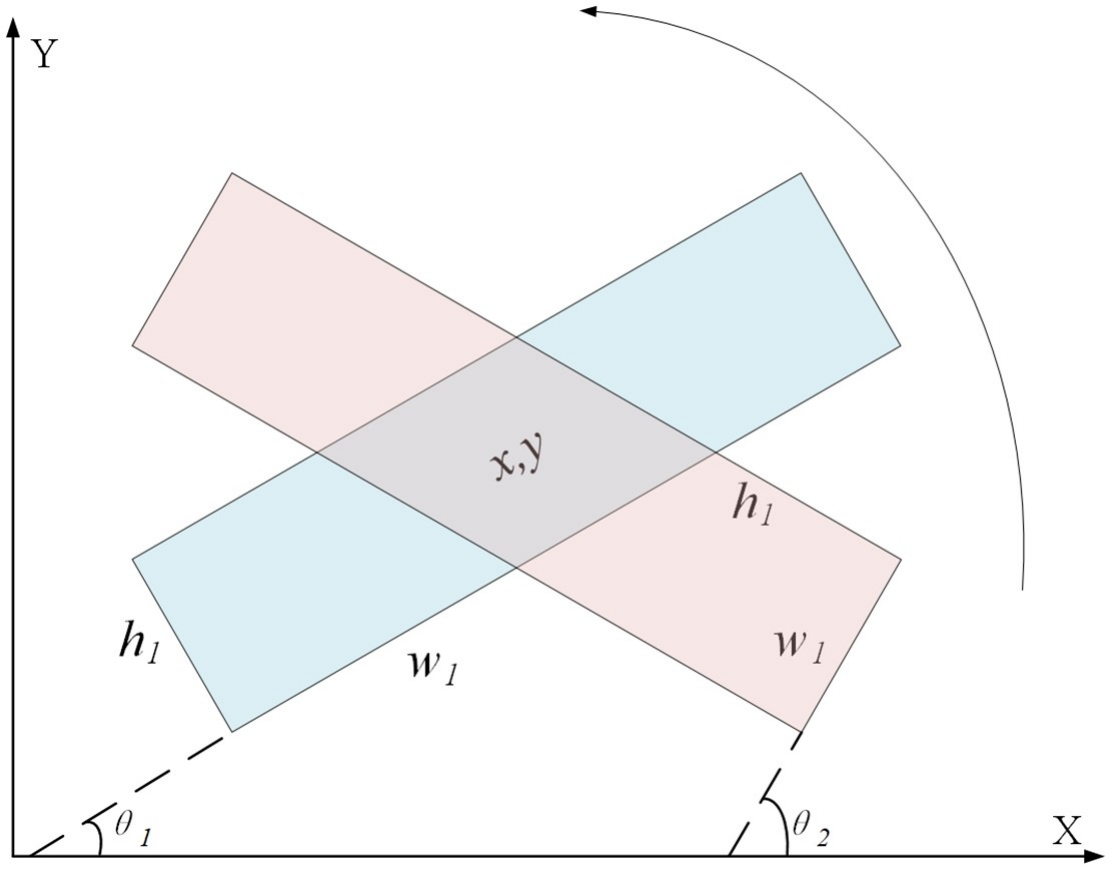
Rotation angle diagram of rotating anchor frame.

Therefore, when two adjacent devices are tilted, the background area will increase and overlap, which will affect the detection effect. For this problem, we propose a rotating anchor scheme with two parameters of aspect ratio and angle to generate anchors. The rotation anchor scheme does not apply the coordinate method of two points, but the five parameters (x, y, w, h, *θ*) of the centre point and the width and height to represent the inclined bounding box, where (x, y) are the centre point coordinates, w and h are width and height, and *θ* is the angle of inclination. As shown in Fig 5, the centre point coordinates (x, y) represent the detection box, and *θ* is the angle between the horizontal axis (X axis) and the first side encountered by the horizontal axis when it rotates counter-clockwise. Meanwhile, this side can be defined as w and the other side as h. It can be seen that *θ*1 and *θ*2∈(0,π/2].

**Fig 6:**
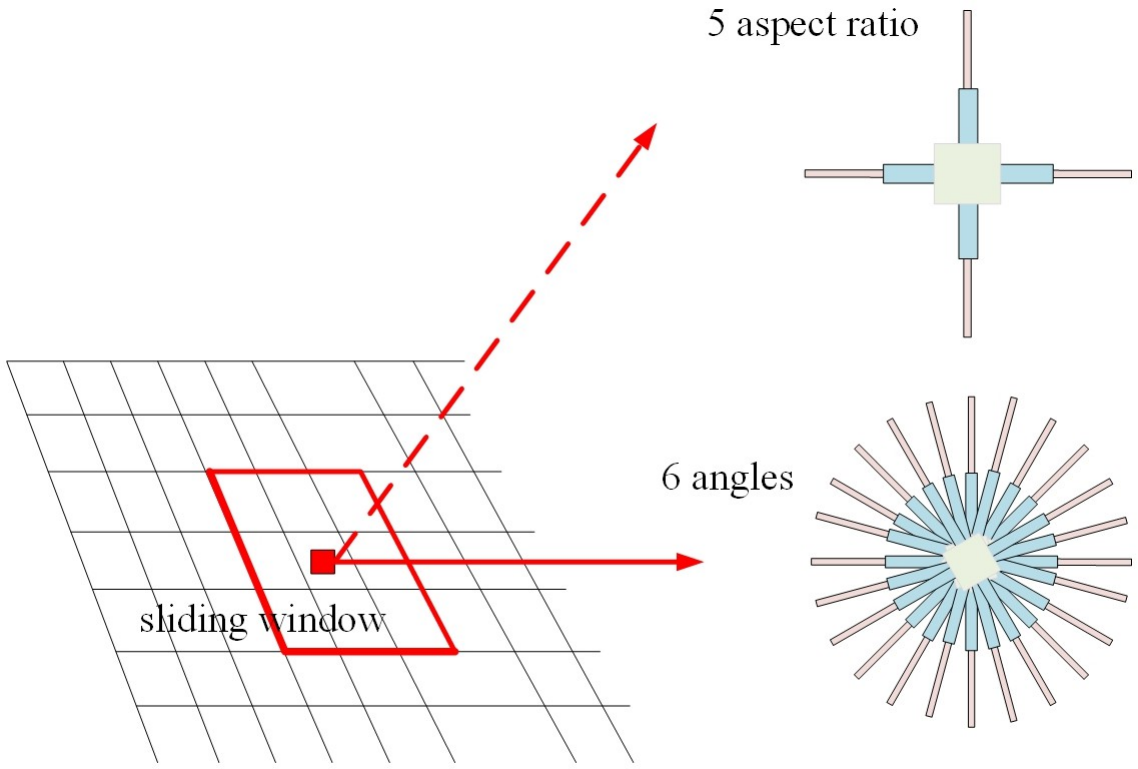
Schematic of the specific rotation anchor.

Five kinds of aspect ratio information were obtained by k-means clustering algorithm, and six angles of information including π/12, π/6, π/4, π/3, 5π/12, π/2 were set at the same time. Therefore, each feature point of each feature map can generate 30 (5 aspect ratio *6 angles) anchors, each classification layer has 30*2 parameter outputs, each regression layer provides 30*5 parameter outputs. Meanwhile, for each output layer of the MobileNetV2 FPN network, the anchor scale is set as (64^2^, 128^2^, 256^2^, 512^2^), so as to deal with the problems of different industrial equipment with different scales, different length-width ratios and different degrees of tilt. The generation of the anchor is shown in Fig 6.

### 3.4 Multi-scale RoI poolings

In the RoI Pooling stage, obtaining fixed-size feature map plays a key role in the classification and regression. For each RPN proposal, we choose to increase two pooled sizes, 3*11 and 11*3 for the improved adaption of different industrial equipment with a wide range of aspect ratios in the data set. In contrast to the effect of the original 7*7 pooled size on industrial equipment objects with similar length and width, the sizes of these two pool test the industrial equipment with a large gap between length and width more accurately.

### 3.5 Multi-stage penalty of non-maximum suppression algorithm

The traditional IoU is calculated on two rectangular boxes. Their overlapping part must be rectangular, but when the rectangular box becomes oblique, that is, when *θ* is not equal to 0, their overlapping part may become irregularly shaped polygons. Therefore, this will lead to inaccurate IoU calculations. We use R-IoU, a calculation method for rotating IoU proposed in the literature [20]. By triangulating the polygon, the area of each part can be calculated and then accumulated to achieve improvement in both simplifying complexity and accuracy of the calculation.

Although the anchor scheme is conducive to the improvement of detection accuracy, a large number of anchor boxes will also be generated, leading to the repeated detections, while ordinary Non-Maximum Suppression (NMS) algorithm will cause missed or false detection due to improper threshold setting or inadaptability to the data set.

Therefore, we proposed a Multi-Stage Penalty of Non-Maximum Suppression (MSP NMS) algorithm. It selects punishment factors according to the IoU of any anchor boxes bi and the anchor boxes M with the highest confidence. Instead of directly excluding boxes that overlap with the selected boxes more than a certain threshold so as not to delete too many boxes that are positioned correctly in crowded situations, the penalty factor is used to reduce the confidence of the anchor box, and the larger the IoU between windows, the heavier the penalty. The penalty factor function is shown in Eq (2).


$$Si=\left\{ \begin{array}{l} \text{IoU}\sqrt{1-{\left(M,bi\right)}^{2}},\text{IoU}(M,bi)<0.2\\ (1.106-0.633\text{IoU}(M,bi)),0.2\leq \text{IoU}(M,bi)\leq 0.8\\ \sqrt{1-\text{IoU}{\left(M,bi\right)}^{2}},0.8<\text{IoU}(M,bi) \end{array}\right.$$ (2)

The experiments show that the algorithm works best when the threshold value is 0.2 and 0.8. When the IoU is greater than the threshold value of 0.8, there is a clear tendency to repeat the same object, so its confidence should decay faster; when the it is less than the threshold value of 0.2, there is a tendency not to be the same object, so its confidence should decay more slowly; when IoU is between 0.2 and 0.8, the linear conventional attenuation of confidence is made.

**Fig 7:**
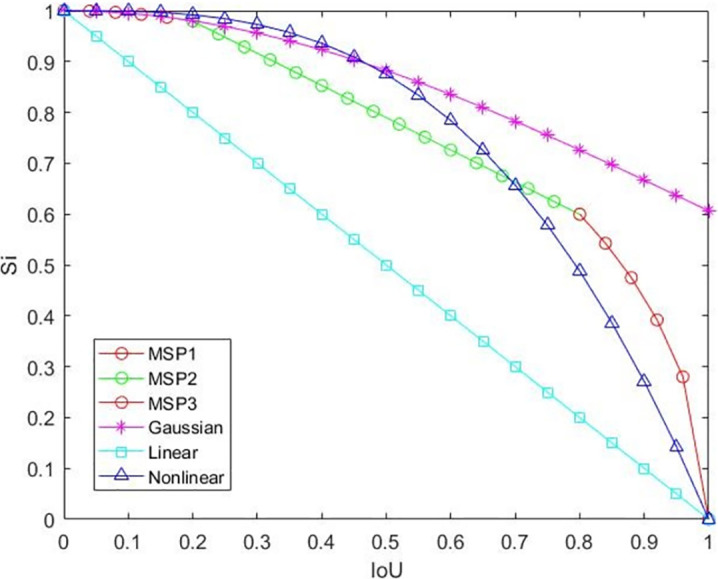
Function graphs of different penalty factors.

Fig 7 shows the function curves of various punishment factors, among which MPS1, MPS2 and MPS3 are the multi-stage punishment factors in the paper, and Gaussian is a Gaussian curve with mean 0 and variance 1. It is obvious that the penalty factor of the linear curve keeps going down fast all the time. The penalty factor is still very high and the multi-stage and the nonlinear punishment factors in this paper are more reasonable. However, the multi-stage punishment factors are taken into account when this tendency of IoU is being too large or small, so the effect is improved.

### 3.6 Multi-task loss function

In order to train RPN, the positive and negative of anchor are redefined here. The conditions for a positive anchor are the IoU of the anchor box and the ground truth box is greater than 0.6 and the angle difference is less than π/12. Or the IoU of the anchor box and the ground truth box is the largest. The negative anchor conditions are the IoU of the anchor box and the ground truth box is greater than 0.6, but the angle difference is greater than π/12. Or the IoU of the anchor box and the ground truth box is less than 0.25. In the remaining cases, the anchor will be unmarked or discarded directly. The loss function plays a key role in model training. The multi-task loss function of ROMS R-CNN is shown in Eq (3).


$$L\left(p_{i},n_{i},l_{i},l_{i}^{*}\right)=\frac{1}{N_{cls}}\sum_{i}L_{\;cls}(p_{i},n_{i})+\xi \frac{1}{N_{reg}}\sum_{i}n_{i}L_{\;reg}(l_{i},l_{i}^{*})$$ (3)

Where *n*_*i*_ represents the indicating value of foreground or background for each anchor (*n* = 1 represents the object area, *n* = 0 represents the background area). *p*_*i*_ stands for the probability of the classification calculated by Softmax. The predicted box and the ground truth box with a positive anchor are respectively represented by *l*_*i*_ and *l*_i_*, which have five parameters. *N*_*cls*_ and *N*_*reg*_ are used for standardization. *ξ* is used to regulate the weighting of two losses. *L*_*cls*_ is logarithmic loss, and *R* in *L*_*reg*_ is smooth *L*_1_ function. The regression loss is activated only for positive bracings (*n* = 1), as shown in Eqs (4)–(9).


$$L_{cls}(p,n)=-\text{log}\;np$$ (4)

$$L_{reg}(l_{i},l_{i}^{*})=R(l_{i}-l_{i}^{*})$$ (5)

$$\text{smooth}\;L_{1}(x)=\left\{\begin{array}{ll} 0.5x^{2} & if\left|x\right|<1\\ \left|x\right|-0.5 & otherwise \end{array}\right.$$ (6)

$$l_{i}^{*}={\left\{l_{x}^{*},l_{y}^{*},l_{w}^{*},l_{h}^{*},l_{\theta }^{*}\right\}}_{i}\;l_{i}={\left\{l_{x},l_{y},l_{w},l_{h},l_{\theta }\right\}}_{i}$$ (7)

For bounding box regression, x, x_a_ and x*are for the predicted box, anchor box, and ground-truth box respectively. The same is true for y, w, and h. The calculations for *l*_*i*_ and *l*_i_* are shown below, and the parameter *k* is used to keep the parameter *θ*∈(0, π/2].


$$l_{x}=(x-x_{a})/w_{a}\;l_{y}=(y-y_{a})/h_{a}\;l_{w}=\text{log}(w/w_{a})\;l_{h}=\text{log}(h/h_{a})\;l_{\theta }=\theta -\theta_{a}+k\pi /2$$ (8)

$$l_{x}^{*}=(x^{*}-x_{a})/w_{a}\;l_{y}^{*}=(y^{*}-y_{a})/h_{a}\;l_{w}^{*}=\text{log}(w^{*}/w_{a})\;l_{h}^{*}=\text{log}(h^{*}/h_{a})\;l_{\theta }^{*}=\theta^{*}-\theta_{a}+k\pi /2$$ (9)

## 4 Experiment and analysis

### 4.1 Data set processing

The data set consists of three categories of industrial equipment, including valves, insulators, and level gauges. The data set is expanded by means of mirror symmetry, cropping, rotation and other data enhancement methods, and 1314 images are produced. Due to the angle information of tilting industrial equipment, we manually label them with visual image calibration tool software. The data format with four-point coordinates is labelled, and then the four-point data set is converted to a format with five parameters of center point coordinates, length, width, and angle using the forward convert function to achieve an XML tag file compliant with the PASCAL VOC standard format. Classification and location information can be quickly viewed in excel, and eventually converted to data sets in tfrecord format.

### 4.2 Experimental environment and parameters

In order to evaluate the feasibility of detecting conditions for industrial equipment, according to the limitation of computing capacity of industrial equipment in actual factories, we use low-profile processors to train and test the detection model. Experimental hardware environment GPU is a NVIDIA MX250. This kind of hardware industrial equipment can be realized in every actual factory. The software operating system is Linux ubuntu16.04, and the training parameters of the experiment are shown in Table 3.

**Table 3:** Parameter setting table for training feature extraction network.

The parameter name	The parameter value
BASE_LR	0.002
BATH SIZE	1
WEIGHT DECAY	0.0001
MOMENTUM	0.9
SAVE_WEIGHTS_INTE	1000
SHOW_TRAIN_INFO_INTE	20

BASE_LR represents the basic learning rate, which is set according to the training effect experience. BATH SIZE represents the batch quantity, which is limited by GPU computing power. WEIGHT_DECAY can decay weights. At the same time, the model overfitting can be reduced to some extent. Momentum represents the effect of the previous iteration on the next iteration in the training process. Increasing the Momentum value can accelerate the iteration speed, but if the Momentum value is set too large, the iteration loss will be increased. SHOW_TRAIN_I NFO_INTE and SAVE_WEIGHTS_INTE represent the display and save of training, which need to be adjusted according to the training situation.

### 4.3 Convolution visualization

**Fig 8:**
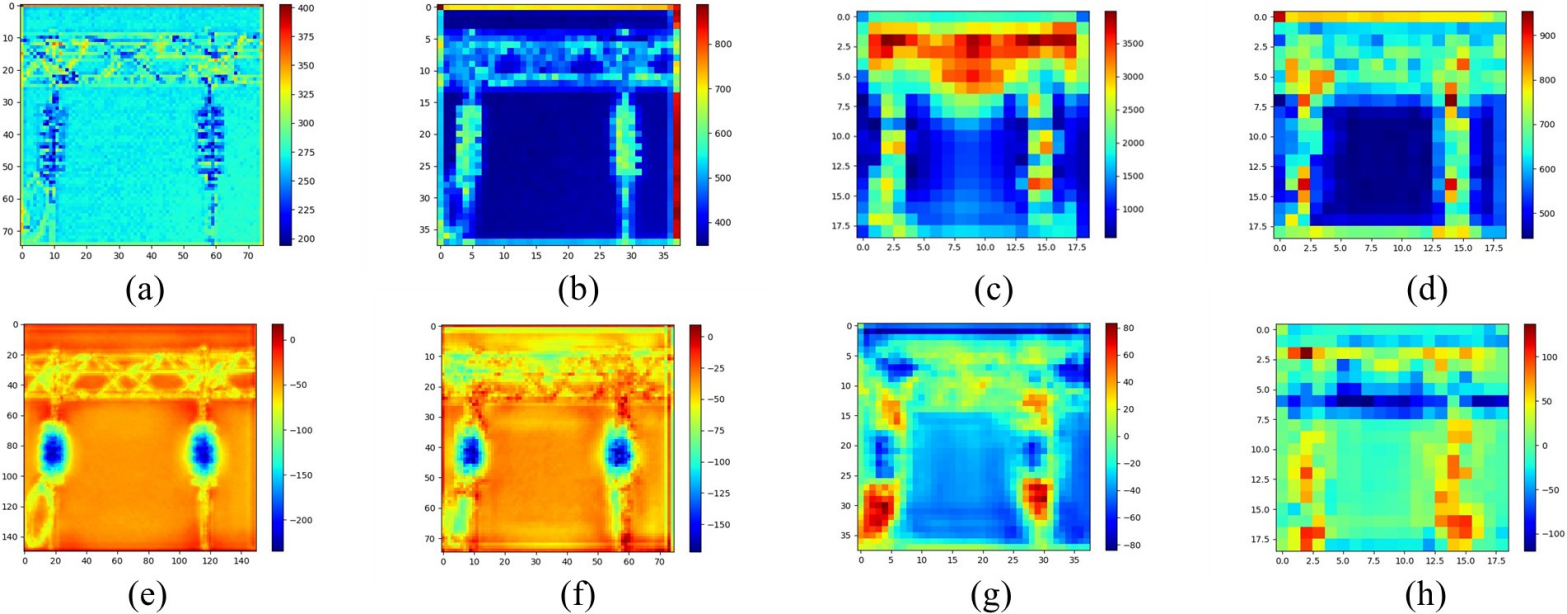
Visualization of convolution process in model training. (a)C2 layer (b)C3 layer (c)C4 layer (d)C5 layer (e)P2 layer (f)P3 layer (g)P4 layer (h)P5 layer. Fig (a) to (d) is the process of down-sampling the feature map. As the down-sampling progresses, the detailed features of the image begin to become blurred, and the semantic features are enhanced. Fig (h) to Fig (e) is the process of sampling on the feature map. With the fusion of P5C5, P4C4, P3C3, P2C2 to P1C1, the semantic features of the feature map are enhanced while the detailed features are also improved. In the end, we got a feature map with strong semantic features and detailed features in the P2 layer.

The processes of network training and feature extraction on insulators of industrial equipment can be observed through convolution visualization. Fig 8 shows the feature extraction of each layer of the MobileNetV2 FPN network.

### 4.4 Performance analysis

The P-R curve describes the relationship between precision and recall. Precision refers to the proportion of true cases, in which all predictions are positive. Recall rate refers to the proportion of all positive cases predicted to be true cases. As shown in Eqs (10) and (11).

$$\text{Precision}=\frac{\text{TP}}{\text{TP+FP}}$$ (10)

$$\text{Recall}=\frac{\text{TP}}{\text{TP+FN}}$$ (11)

where TP is the number of samples in which the positive cases are predicted as positive, FP is the number of samples in which the negative cases are predicted as positive, and FN is the number of samples in which the positive cases are predicted as negative. Average Precision (AP) and mean Average Precision (mAP) can well evaluate the detection performance after training. Its calculation is shown in Eqs (12) and (13).


$$\text{AP}=\int_{0}^{1}p(r)dr$$ (12)

$$\text{mAP}=\frac{1}{N_{cls}}\sum_{i}{\text{AP}}_{i}$$ (13)

**Fig 9:**
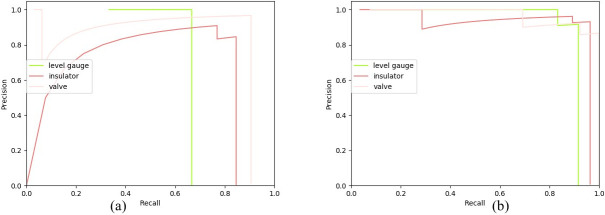
P-R curve contrast. (a) P-R curve of Faster R-CNN (b) P-R curve of ROMS R-CNN. It represents the AP comparison curve using two algorithms Faster R-CNN and ROMS R-CNN to detect the three categories of liquid level gauge, valve and insulator.

Fig 9 show that the AP of various types in ROMS R-CNN in (b) is superior to the Faster R-CNN in (a). We can also get that the AP using algorithm ROMS R-CNN is greater than the AP using algorithm Faster R-CNN.

We conducted a comparative experiment on ROMS R-CNN and ROMS R-CNN 1–3, and the results are shown in Table 4. It can be found that the mAP is constantly improved, which indicates that each structure proposed in this paper plays a role in improving the detection accuracy. Compared with ROMS R-CNN-1 and ROMS R-CNN-2, it can be found that the AP of the valve has a slight downward fluctuation, and the AP of the insulator and the liquid level gauge have an increase of 0.051 and 0.099 respectively, which indicates that the detection effect of the liquid level gauge and the insulator can be improved by the specific rotating anchor scheme. It has no effect on valve testing because the circular structure of the valve does not have the problem of rotation tilt. Compared with ROMS R-CNN-2 and ROMS R-CNN-3, similar results can still be found, but the multi-scale RoI pooling makes the AP elevation of insulator and liquid level meter become limited, increasing by 0.01 and 0.023, respectively. Finally, by comparing ROMS R-CNN-3 with ROMS R-CNN, it was found that the AP of valve and insulator is increased by 0.012 and 0.018 respectively. The MSP NMS algorithm has obvious effect, but it has no effect on the liquid level gauge. There is littler mutual occlusion because the slender structure of the liquid level gauge removes a large number of irrelevant backgrounds from the candidate box after passing through the rotating anchor scheme. In addition, due to the characteristics of industrial production, there are few adjacent liquid level gauges, so the MSP NMS algorithm doesn’t improve the performance.

**Table 4:** mAP comparison of three kinds of industrial equipment detection by ROMS R-CNN algorithm with different structures and functions.

Method	MobileNetV2FPN	Special Rotation Anchor	Multi-scale RoI poolings	MSP NMS	AP			mAP
					Valve	Insulator	Level gauge	
**ROMS R-CNN-1**	√	×	×	×	0.959	0.857	0.790	0.869
**ROMS R-CNN-2**	√	√	×	×	0.957	0.908	0.889	0.918
**ROMS R-CNN-3**	√	√	√	×	0.960	0.918	**0.912**	0.930
**ROMS R-CNN**	√	√	√	√	**0.972**	**0.936**	0.910	**0.939**

We also conducted comparative experiments on ROMS R-CNN and other algorithms. In most of the comparable algorithms, YOLO algorithm shows lower accuracy and faster speed in contrast to R-CNN algorithm. However, in the actual application of industrial equipment inspection, due to the priority on safety, stability and higher requirements for accuracy, R-CNN algorithm is selected as the basis. In this paper, according to the multi-scale and rotation-tilt characteristics of industrial equipment, FPN with better multi-scale detection effect and RRPN algorithm which can perform rotation detection are respectively selected and compared with the algorithm in this paper. The results are shown in Table 5. Comparing Faster R-CNN, FPN, and ROMS R-CNN-1, we can find that the effect of FPN network is quite obvious, and the improvement of AP is relatively great, but the performance of our ROMS R-CNN-1 is slightly lower than FPN due to the adoption of the network structure of ResNet-101 in FPN. The MobileNetV2 is a lightweight network, which is more suitable for mobile industrial equipment detection and more in line with the actual conditions of industrial equipment detection. From Table 5, we can see that for the detection of industrial equipment, our ROMS R-CNN is better than other algorithms, and the mAP reaches 0.939.

**Table 5:** mAP comparison table of three kinds of industrial equipment detected by different algorithms.

Method	Network Model	Pool Size	NMS Algorithm	Method of anchor	AP			mAP
					Valve	Insulator	Level gauge	
**Faster R-CNN**	VGG-16	7*7	Ordinary NMS	Ordinary anchor	0.890	0.797	0.735	0.808
**FPN**	ResNet-101	7*7	Ordinary NMS	Ordinary anchor	0.971	0.874	0.803	0.883
**RRPN**	ResNet-50	7*7	RNMS	R-Anchor	0.901	0.866	0.854	0.874
**ROMS R-CNN**	MobileNetV2 FPN	7*7 3*1111*3	MSP NMS	Special Rotation Anchor	**0.972**	**0.936**	**0.910**	**0.939**

This is the AP result and mAP result of three different industrial equipment detection using four different algorithms.

We used different feature extraction networks in the same operating environment to carry out detection experiments on industrial equipment to compare the time consumption of two feature extraction networks, MobileNetV2 FPN, VGG-16 and ResNet-50, as shown in Table 6. The lightweight detection network is significantly faster than ResNet-50 and VGG-16 due to its greatly reduced number of parameters. Meanwhile, as shown in Table 5, the loss of mAP is low. Therefore, the MobileNetV2 FPN network is more in line with the actual needs of industrial equipment testing.

**Table 6:** Comparison table of average detection time of different network structures.

Model	MobileNetV2 FPN	VGG-16	ResNet-50
**Average detection time**	0.435s	0.459	0.614s

This is the average time of industrial equipment detection using different feature extraction models MobileNetV2 FPN, VGG-16 and RESNET-50 detection algorithms.

We compared the actual detection effect between ROMS R-CNN algorithm (green detection box) and Faster R-CNN algorithm (red detection box). Since the prediction box of ROMS R-CNN algorithm is not always horizontal, the confidence is marked at the centre point.

**Fig 10:**
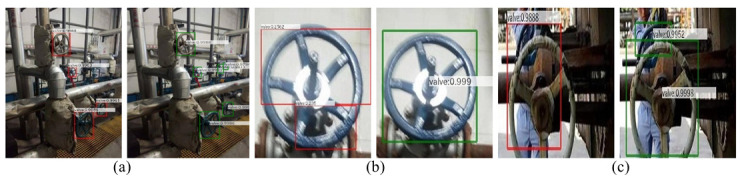
Comparison diagram of valve detection. (a) Small scale valve detection (b) Large scale valve detection (c) Occlusion overlap valve detection. In Fig (a), (b) and (c), the left-hand picture is the detection result of the Faster R-CNN algorithm, and the right-hand picture is the detection result of the ROMS R-CNN algorithm. The label in the figure marks the name and confidence of the object. In Fig (a), there are two small scale valves. The left picture fails to be detected, resulting in missed detection, and the right picture successfully detected. In Fig (b), the valve scale is too large. On the left, one valve is detected as two, resulting in false detection. On the right, large-scale valves are successfully detected. In Fig (c), the valves are overlapped seriously with each other.

The comparison of valve detection is shown in Fig 10. Tilt detection is almost non-existent due to the geometry of the valve with different size, covering or overlapping each other. It can be seen that the detection effect of Faster R-CNN algorithm is poor, with missed detection and misdetection occurring in every case, while our ROMS R-CNN algorithm can detect industrial equipment in the case of multi-scaled and overlapping occlusion.

**Fig 11:**
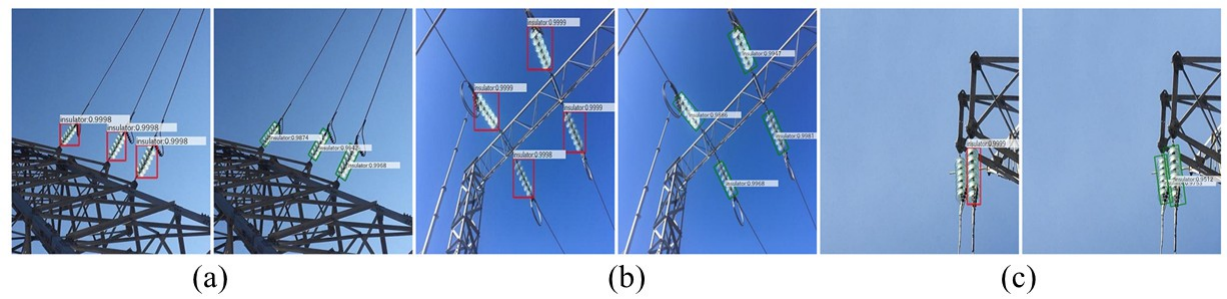
Comparison diagram of insulator detection. (a) left tilt detection (b) right tilt detection (c) occlusion overlap detection. In Fig (a), (b) and (c), the left-hand picture is the detection result of the Faster R-CNN algorithm, and the right-hand picture is the detection result of the ROMS R-CNN algorithm. The insulator in Fig (a) tilts to the left. In Fig (b), the insulator tilts to the right. The prediction box on the left is horizontal and the prediction box on the right is tilted. In Fig (c), insulators are slightly shielded. One insulator object is missing in the left picture, and two insulator objects are successfully detected in the right picture.

The comparison of insulator detections is shown in Fig 11, where the insulator is tilted to the left or right and slightly blocked. The prediction box of the insulator in the Faster R-CNN algorithm is horizontal and contains a large number of unrelated background regions, which may be missed due to overlap of occlusion. However, our ROMS R-CNN algorithm has a better detection effect because the prediction box is tilted and there is almost no background area. It can not only detect devices tilted in different directions, but also avoid missed detection caused by overlap of occlusion.

**Fig 12:**
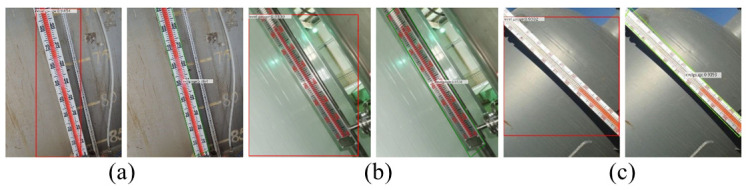
Comparison diagram of level gauge detection. (a) small angle tilt detection (b) moderate angle tilt detection (c) large angle tilt detection. In Fig (a), (b) and (c), the left-hand picture is the detection result of the Faster R-CNN algorithm, and the right-hand picture is the detection result of the ROMS R-CNN algorithm. In Fig(a), the tilt angle of the level gauge is small. The tilt angle of the level gauge in Fig(b) and (c) gradually increases. The prediction box in the left picture contains a large number of irrelevant background regions, and the bounding box regression effect is not ideal. The skew prediction box on the right has removed the irrelevant background area.

The comparison of level gauge detection is shown in Fig 12, where the inclination angle of the level gauge gradually increases. The prediction box of the Faster R-CNN algorithm contains a large number of irrelevant background areas, and the bounding box regression effect is not ideal, while the oblique prediction box of our ROMS R-CNN algorithm removes the irrelevant background area. The effects of industrial equipment detection with different rotation and tilt angles are improved.

## 5 Conclusions

This paper proposes a ROMS R-CNN industrial equipment detection method, which adopts the optimization algorithms such as MobileNetV2 FPN, Specific Rotation Anchor, Multi-scale RoI poolings, MSP-NMS, etc. The industrial equipment under complex working conditions, such as multi-scale, rotation tilt, overlapping and shielding, was successfully detected through the processes of data collection, production, model training and optimization, and actual detection. Experimental results show that the proposed method increases mAP from 0.797 to 0.939, effectively reducing the rate of false detection and missed detection.
